# Supplementary material for: Assessment of multiple herbicide protection seed treatments for seed-based restoration of native perennial bunchgrasses and sagebrush across multiple sites and years
Source: PLoS One. 2023 Mar 30;18(3):e0283678. doi: 10.1371/journal.pone.0283678 (PMC10062626; doi:10.1371/journal.pone.0283678)
Supplement: S1 Appendix — (DOCX) [file pone.0283678.s001.docx]

**S1 Appendix. Additional methods**

**Seed lot selection**

In 2018 and 2019, we followed empirical or provisional seedzones [1] to most appropriately match seed sources to each site, generally resulting in different seed sources for each species seeded at each site in those years (S2 Fig, S1 Table 1). In 2020, to reduce experimental complexity when working with multiple partner organizations to produce seed treatments, only one seed source of each species was used across all sites, and this source was chosen from the empirical seed zone that either matched or was warmer/more arid than the zones in which the sites occurred.

**S2 Figure. Map of field sites used**. Site names correspond to tables. In Idaho and Wyoming, original sites (1) were relocated to improved sites (2), and trials were never carried out in both sites in the same year within a state.

**S1 Table 1. Seedlot details.**

| **SEEDING YEAR** | **SITE** | **SPECIES** | **SELECTION** | **Lot ID** | **GERMPLASM ORIGIN** | **VIABILITY AT SOWING** | **SEEDS PER BULK GRAM** |
| --- | --- | --- | --- | --- | --- | --- | --- |
| 2018 | OR | ELEL | Deschutes | L&H SIHY-1458-0 | near Prineville, OR | 69% | 271 |
|  |  | POSE | Paulina | BFI-15-10188563 | near Paulina, OR | 65% | 1677 |
|  | NV | ELEL | Sonoma | BFI-16-10130102 | near Winnemucca, NV | 85% | 425 |
|  |  | POSE | Panther | BFI-17-10188103 | near Winnemucca, NV | 96% | 1713 |
|  | WY1 | ARTR | (unamed) | SOS-WY050-150-16 | Central WY | 92% | 3673 |
| 2019 | OR | ELEL | Deschutes | BFI-17-10130515 | near Prineville, OR | 82% | 271 |
|  |  | POSE | Paulina | BFI-16-10188563 | near Paulina, OR | 88% | 1677 |
|  |  | ARTR | Vines Hill | n/a, 2018 harvest | near Vale, OR | 73% | 3290 |
|  | NV | ELEL | Sonoma | BFI-16-10130102 | near Winnemucca, NV | 83% | 425 |
|  |  | POSE | Panther | BFI-17-10188103 | near Winnemucca, NV | 88% | 1713 |
|  |  | ARTR | (unnamed) | BS4016MMWY | near Winnemucca, NV | 86% | 4144 |
|  | ID1 | ELEL | Blue Mt. | BFI-16-10130881 | near Lime, OR | 88% | 270 |
|  |  | POSE | Mt. Home | 8UP154-40 | near Mountain Home, ID | 95% | 1976 |
|  |  | ARTR | Vines Hill | n/a, 2018 harvest | near Vale, OR | 69% | 3290 |
|  | WY2 | ELEL | (unnamed) | 1882-1 | Southern ID | 66% | 151 |
|  |  | POSE | Vale | 1843-0 | Malheur Co., OR | 69% | 1990 |
|  |  | ARTR | (unnamed) | V-109212 | Central WY | 81% | 905 |
|  | UT | ELEL | Pueblo | 1882-1 | near Pueblo, CO | 90% | 141 |
|  |  | POSE | Mt Home | NBS-CF18-MTH-1 | near Mountain Home, ID | 89% | 1907 |
|  |  | ARTR | (unnamed) | V-108884 | Southern ID | 85% | 1164 |
| 2020 | All  sites | ELEL | Turkey Lake | L&H-1934-0 | near Gooding, ID | 95% | 269 |
|  |  | PSSP | Anatone | Granite-1601-1 | near Anatone, WA | 79% | 309 |
|  |  | ARTR | (unnamed) | Premier-470 | near Carey, ID | 51% | 2866 |

**Germinability and seeds per gram calculation for calculating seeding rate and viable seed sown**

Prior to producing seed enhancement technologies or any bare seed samples, germination tests were conducted for each seedlot in 8 dishes of 25 seeds per dish on blotter paper in germination chambers set to species-preferred germination temperatures, as specified in the Native Plant Network Propagation Protocol Database [2]. Seed per bulk gram estimates were conducted on 5 samples of approximately 100 seeds per sample in which bulk samples of each seedlot were weighed to the nearest tenth of a miligram, then counting the number of seeds present in the sample, rinsing off seed treatments if needed with water over sieves. Germinability (%) was multiplied by seeds per bulk gram to attain viable seed (PLS) per bulk gram. Desired seed rate per sample (either 400 or 572 PLS) was divided by PLS per bulk gram to attain the target weight for the sample, and all samples were weighed to nearest tenth of a milligram, and each sample’s exact weight was recorded. Individual sample weights were then used to calculate each sample’s specific PLS rate (viable seeds sown). This sample-specific viable seed sown estimate was used as the denominator for calculation of each subplot’s seedling emergence, expressed as the proportion of viable seed sown that emerged. This process corrects for unavoidable minor variation among samples and sample types (especially for HPPs, whose weight cannot be metered exactly to the sample target weight), ensuring that these small differences in seeding rate are not confounded with treatment effects.

**Site preparation and seeding**

In 2018 and 2019, the experimental setup at each site consisted of a single, rectangular, completely randomized, full factorial design with five replicates, for a total of 140 (2018) and 200 (2019) experimental plots per site. Experimental plots were each 1 m x 1 m, and were separated from each other by at least 1m and no more than 5m on all sides. Litter was reduced on all plots not assigned to the litter intact treatment (2019 only) using rakes. At all sites, raking occasionally produced disturbance to the top layer of the soil at some plots, though this was minimized as possible. In 2020, the experimental setup at each site consisted of seven ~9 m x 7 m split plots (split = herbicide treatment), with each split being 3.5 m x 9 m and containing a single replicate of each of 12 unique treatments in 1 x 0.5 m subplots, totaling 168 subplots per site. In this design, subplots were immediately adjacent to one another on the short end in two rows of 6 subplots, with 0.5 meters between rows. Litter was not reduced or modified in 2020.

For subplots receiving the shallow furrow seed delivery treatment (half of all plots in 2018 and 2019, all subplots in 2020), 0.75 – 1.0” deep, 1 – 2” wide furrows were made by hand with a 0.3" thick steel blade, 8 inches apart, perpendicular to the slope angle. Furrows were made no more than 24 hours prior to seeding. Subplots were then uniformly seeded by hand, either in the furrow only, or over the entire subplot area for the broadcast treatment (2018 and 2019 only). Across all sites and years, seeding occurred mostly in October, though sometimes as early as September or as late as November as a result of logistical costraints (S1 Table 2).

**S1 Table 2.** **Dates for experimental activities.**

|  |  |  |  | **MONITORING** | | **GERM BAG HARVESTS** | | |
| --- | --- | --- | --- | --- | --- | --- | --- | --- |
| **SITE** | **YEAR** | **SEEDED** | **SPRAYED** | **EARLY*** | **LATE** | **START WINTER**** | **START SPRING** | **END SPRING** |
| OR | 2018 | 10/30/18 | | 4/8/19 | 5/14/19 | 12/28/18 | 3/11/19 | 5/14/19 |
| NV |  | 11/28/18 | | 4/23/19 | 5/28/19 | 1/11/19 | 3/10/19 | 4/24/19 |
| WY1 |  | 11/15/18 | | 4/13-15/19 | 6/25-27/19 | 1/10/19 | 3/25/19 | 5/18/19 |
| OR | 2019 | 11/11/19 | 11/12/19 | - | 05/17-19/20 | 1/5/20 | 2/23/20 | 5/19/20 |
| NV |  | 10/16/19 | 10/18/19 | - | 05/28-29/20 | 1/10/20 | 3/9/20 | 5/29/20 |
| UT |  | 10/31/19 | 11/1/19 | - | 05/26-27/20 | 1/9/20 | 3/5/30 | 5/27/20 |
| WY2 |  | 10/22/19 | 10/23/19 | - | 06/03-04/20 | 1/3/20 | 3/9/20 | 6/4/20 |
| ID1 |  | 10/23/19 | 10/24/20 | - | 05/26-27/20 | 1/1/20 | 2/12/20 | 5/27/20 |
| OR | 2020 | 10/19-20/20 | 10/21/20 | 4/5-7/21 | 5/3-4/21 | - | 2/23/21 | 5/3/21 |
| NV |  | 10/5-6/20 | 10/7/20 | 4/12-14/21 | 5/10-12/21 | - | 3/8/21 | 5/12/21 |
| UT |  | 10/5-6/20 | 10/8/20 | 4/12-13/21 | 5/13-14/21 | - | 3/5/21 | 5/14/21 |
| WY2 |  | 09/28-29/20 | 10/1/20 | 4/22-26/21 | 5/19-20/21 | - | 3/17/21 | 5/20/21 |
| ID2 |  | 10/15/20 | 10/16/20 | 4/19-21/21 | 5/17-19/21 | - | 3/2/21 | 5/19/21 |

*COVID-19 pandemic prevented early site visit
**Only two germination bag harvests were conducted in 2020 due to logistical constraints

Within 1-2 days of seeding, a single application of herbicide solution was applied with a backpack sprayer to all subplots receiving the herbicide treatment. In 2018 and 2019, single 1 x 1 m subplots were individually sprayed, with approximately 0.5 m overspray on all sides. In 2020, herbicide was applied to each entire 3.5 m x 9 m split, producing a 1 m overspray on all sides of subplots. Herbicide was applied at the target rate of 584 ml/ha (8 oz/ac) formula of the pre-emergent Plateau (137 ml/ha AI of imazapic salt) in the 2018 seeding year, and 730 ml/ha (10 oz/ac) formula of Plateau (172 ml/ha AI) and, for some sites and years, 438 ml/ha (6 oz/ac) formula of the nonselective broadleaf Accord (219 ml/ha AI glyphosate) was added to the same mix to target occasional green-up of some target weed species. In ID in 2019, mixing errors occurred which resulted in a rate of 3.14 l/ha formula of Plateau (741 ml/ha AI). Nothing was applied to plots not receiving the herbicide treatment. All sites experienced no precipitation between seeding and spraying, and at least 12 hours of dry weather immediately after herbicide application.

**Assessment of field germination using buried seed bags**

We employed germination bags to estimate germination rates of bare seed for each seeded species. Germination bags were 4”x 6” nylon organza bags, containing either 50 (2018) or 100 (2019, 2020) seeds per bag, and were sown simultaneously with seeded plots as shallow as possible (0.25” to 0.5”) beneath unsifted field soil. In 2018, 200g of fine, site-specific field soil was included in each bag to improve seed-soil contact, but soil was omitted from bags in 2019 and 2020 to promote shallower seed burial. Two or three harvest dates were used: beginning of winter (2018, 2019; typically mid-late December or early January), beginning of spring (all three years; typically in late February or March), as well as end of spring (all three years, late May). Target dates for the beginning of winter and spring at each site were set by examining modeled average soil temperatures at 2 cm depth using the SHAW model [1].

Beginning of winter was defined as no sooner than each site’s 2 cm soil temperatures bottomed out or dropped below 0°C for more than 75% of each day; typically in mid-late December or early January. Beginning of spring was when 2 cm soil temperatures began to rise above 0°C for a majority of the day, typically in late February or March. End of spring was no sooner than when 2 cm soil temperature remained above 0°C for the duration of the growing season, and was typically in late May. Site-local weather conditions and forecasts were assessed prior to each harvest to confirm the above criteria, and harvest dates were either delayed or expedited accordingly.

Harvested bags were immediately frozen if harvested wet, or immediately air-dried if harvested dry. They were processed by rinsing soil and detritus from sealed bags, then carefully cutting edges from bag, turning bag inside out over a sieve and recovering all seed. Each seed was then scored as germinated or not. Sagebrush seed often sheds its dark-colored seed coat when germinated, and early-germinating seedlings that subsequently died often dissolved completely, leaving only this empty seed coat; therefore, these empty coats were scored as germinated seed when they were encountered.

**Data collection**

Density of seeded species were measured in the entire seeded subplot area for every sampling event, including any individuals of those species recruiting naturally in the seeded or unseeded plots (which were never common; see below). Emerged seedlings found during early sampling events were marked with colored toothpicks, so that survival of this cohort could be assessed at the late event, and so that we could better measure cumulative emergence over the entire season. All emergence densities were divided by the number of viable seeds sown for that seed treatment and subplot before analysis, to generate a more standardized response to compare seed treatment efficacy, even if seeding rates varied slightly. Seeded species height and leaf count were measured in the late sampling only, and were taken on the three seedlings in each subplot closest to a consistently marked point on sampling frames, and then averaged for that subplot before analysis.

Density of non-seeded, onsite vegetation was assessed in the entire seeded area unless densities were so high that counts would regularly be in the hundreds, in which case the sampling area for that entire site and sampling event were downscaled to a portion of the seeded area that would regularly produce less onerous counts. These downscaled counts were later scaled back up to the full seeded area before analysis. Percent foliar cover per group was visually estimated to the nearest percent across the entire seeded area for every group.

**Data transformation and analysis**

In 2018, a square root transformation was used for early and late seedling count, and density of IAG and IAF for both early and late counts, as this transformation improved residual normality for all. In 2019, densities per square meter of IAG, IAF, OS, and OA were cube-root transformed, and all other responses were square-root transformed, with all transformations improving normality of model residuals. In 2020, percent emergence of seeded grass and shrub species early and late both showed non-normal distributions of full-model residuals, and were transformed using arcsin (sqrt(x+0.002)) to improve normality. Similarly, densities of IAG, IAF, OA, and OS were also transformed to improve model residuals, using a Log10(x+1) transformation.

 In all years, data from unseeded plots were examined for notable densities of resident individuals of seeded species. Fewer than 5 seedlings of this nature were found at any site in any year, suggesting background establishment of these species was low. Therefore, data from unseeded subplots were removed from analyses of seeding responses, and no corrections to seeded plots were applied to account for background establishment. This data was however included in the 2019 analysis of the effects of litter reduction on herbicide application described above.

**References**

1. United States Department of Agriculture Forest Service; Western Wildland Environmental Threat Assessment Center: TRM Seed Zone Applications. [cited 21 May 2018; 23 June 2020]. Available: https://www.fs.usda.gov/wwetac/threat-map/TRMSeedZoneMapper.php
2. Native Plant Network; Reforestation, Nurseries, and Genetic Resources: Propagation Protocol Database. [cited 06 Jun 2018, 09 July 2020]. Available: https://npn.rngr.net/propagation
3. Flerchinger GN, Caldwell TG, Cho J, Hardegree SP. Simultaneous Heat and Water (SHAW) Model: Model Use, Calibration, And Validation. Trans ASABE. 2012;55: 1395–1411.
