# Supplementary material for: Assessment of multiple herbicide protection seed treatments for seed-based restoration of native perennial bunchgrasses and sagebrush across multiple sites and years
Source: PLoS One. 2023 Mar 30;18(3):e0283678. doi: 10.1371/journal.pone.0283678 (PMC10062626; doi:10.1371/journal.pone.0283678)
Supplement: S2 Appendix — (DOCX) [file pone.0283678.s002.docx]

**S4 Appendix. Additional results.**

**Summary of weather trends**

**S3 Fig. Precipitation and temperature patters for experimental trials**. Mean temperature anomaly (black needles) and precipitation anomaly (hashed bars) for all tested sites (across top) and planting years (2018, 2019, 2020; top to bottom), for winter (Dec – Feb) and spring (Mar – May) seasons, calculated against 1991 – 2020 climate normals. Values are percent deviation from the normal precipitation (left axis) and degrees Celsius (right axis). Data are from PRISM Climate Group.

**Herbicide effects on IAG and IAF density and cover:**

Effects of herbicide application on foliar cover (not taken in 2018 trials) of IAG and IAF was generally a sharp reduction, but varied by year and site. In 2019, herbicide application reduced IAG cover by 62%-100% (average 87%) across all sites (SITE*HERB interaction; F_4,928_ = 429, *P* < 0.001), but only reduced IAF cover in NV (99%) and increased IAF cover in OR (2.8-fold), with no effects at other sites (SITE*HERB interaction; F_4,928_ = 359, *P* < 0.001). In 2020, herbicide application reduced IAG cover by 67% (UT) and 93-99% (all other sites; SITE*HERB interaction; F_,4,774_ = 35.1, *P* < 0.001), and reduced IAF cover by 46% to 100% at all sites in which IAF were present (all but WY; SITE*HERB interaction; F_4,774_= = 122, P<0.001).

The patterns shown by plant density of IAF and IAG (reported in the main manuscript) were regularly tracked by patterns in foliar cover estimates of those plant categories (reported above), except in a few instances. In 2019 herbicide application resulted in increased densities of IAF in UT, decreased densities in ID, and no effect in OR, whereas cover estimates showed no effect in UT or ID, and an increase in OR. Additionally, in 2020, herbicide application had no effect on IAG density at the UT site, but resulted in a 67% reduction in foliar cover.

**Herbicide effects on bare seed success**

There were several interactions involving HERB at OR in 2018 for seeded species late season seedling count and seedling height (see Results; S6 Fig), but neither demonstrated an effect of herbicide treatment. For both, Tukey HSD tests performed on the interactions revealed no differences between levels of HERB among any of the four SPEC*DELIV pairs (S4 Table 1). Similarly, a SPEC*HERB interaction for late seedling count in 2020 for WY (see results) also showed no differences in either of the three species.

**S6 Fig. Herbicide effects on bare seed seedling size**. Effect of herbicide on mean seedling height (mm) and mean seedling leaf count of seedlings derived from the bare seed (unprotected) seed treatment for all three years. In 2018 (A), all species and both delivery methods (broadcast, furrow) are pooled. In 2019 and 2020 (B), only furrow delivery data are included. Black stars indicate significant effect (P < 0.05), and all other comparisons are not significant. Error bars are standard errs. Too few seedlings for some species in some years resulted in not enough data to make statistical comparisons.

**S4 Table 1. Tukey HSD results for significant interactions of herbicide effects on seedling counts from the bare seed treatment.**

| **Treatment combination** | **2018 OR late count** | | | | **2018 Oregon height** | | |
| --- | --- | --- | --- | --- | --- | --- | --- |
|  | **Tukey HSD** | | | **Least Sq Mean** | **Tukey HSD** | | **Least Sq Mean** |
| POSE,NH,FURROW | A | B | C | 0.1397 |  | B | 18.47 |
| POSE,NH,BROADCAST |  | B | C | 0.0483 |  | B | 21.22 |
| POSE,H,FURROW | A |  |  | 0.2318 |  | B | 18.13 |
| POSE,H,BROADCAST |  |  | C | 0.0200 | A | B | 14.00 |
| ELEL,NH,FURROW | A | B |  | 0.1589 | A |  | 48.47 |
| ELEL,NH,BROADCAST |  | B | C | 0.0400 | A | B | 40.00 |
| ELEL,H,FURROW |  | B | C | 0.0757 | A | B | 34.78 |
| ELEL,H,BROADCAST |  |  | C | 0.0200 | A |  | 61.00 |
|  | **2020 WY late count** | | | |  |  |  |
| PSSP,NH |  | B |  | 0.0447 |  |  |  |
| PSSP,H | A | B |  | 0.0532 |  |  |  |
| ELEL,NH | A |  |  | 0.0763 |  |  |  |
| ELEL,H | A | B |  | 0.0532 |  |  |  |
| ARTR,NH |  | B |  | 0.0424 |  |  |  |
| ARTR,H |  | B |  | 0.0447 |  |  |  |

**Detailed comparison of seeding outcomes (seedling counts and size) among herbicide protection treatments, with and without herbicide**

Results describing all differences in seeding outcomes produced by the various HP treatments tested in each year, with and without herbicide, are provided below, divided by year, and a summarized version is provided in the main text.

**2018 seeding: large vs small pellets**

Differences in seedling count between herbicide treatments varied by site, species, herbicide application, and sampling season. Overall, large and small multi-seed pellets performed similarly, but in a few cases large pellets performed better than small pellets. At the NV site for POSE, large HP pellets had 2.5-fold higher early seedling count than small HP pellets when herbicide was applied, but no such difference in the absence of herbicide (SPEC*HPTRT*HERB interaction; F_2,96_ = 3.1, *P* = 0.05). At the WY site for ARTR, early but not late season seedling count was 1.5-fold higher for large than small HP pellets, regardless of whether herbicide was applied (SPEC*HPTRT interactions; F_2,96_ = 7.7 – 12.1, *P* < 0.001). For all other species and sites (ELEL in NV, and ELEL and POSE in OR) there were no other significant differences in early or late season seeded species seedling count between small and large pellets, whether herbicide was applied or not.

Height and leafiness of seeded species seedlings at OR in the late season were 38-50% greater for large than small pellets regardless of species when herbicide was applied (with no difference in the absence; HPTRT*HERB interactions; F_2,73_ = 3.5 – 5.7, *P* < 0.033). There were no other significant differences among HP pellet sizes at any site for any species.

**2019 seeding: Large vs small pellets (grasses) and small pellets vs AC banding**

For grasses, late season grass seedling count was 2.1-fold higher for large (0.53%) than small pellets (0.26%) in the absence of herbicide, regardless of site or species (HPTRT*HERB interaction; F_2,240_ = 4.22, *P* = 0.0159). In the presence of herbicide, both pellet sizes produced higher mean seedling count than in the absence, but they did not differ from one another. For sagebrush in OR, there were no differences in seedling count between small pellet and carbon strip treatment. The only differences in seedling size measurements between HP seed treatments for any species at any site was for ARTRW in OR in the presence of herbicide, where carbon strip reduced seedling height by 55% compared to small pellet (S7 Fig; HPTRT*HERB interaction, F_2,12_ = 3.90, *P* = 0.0493).

**S7 Fig. HP and bare seed treatment effects on seedling size.** Differences in seedling size (mean height and leaf count) among seed treatments. In 2018 (A), differences among carbon seed treatments were dependent upon exposure to herbicide (top), but notable differences between carbon treatments and bare seed were not (bottom). Black stars indicate significant difference in ANOVA model (P < 0.05). Bars sharing the same letters within each site for 2020 (B) and for each species in 2019 (C) are not significantly different according to post-hoc Tukey HSD tests (P < 0.05).

**2020 seeding: small HP pellet vs vortex coating vs Kamterter coating for grasses, small HP pellet vs slurry for sagebrush**

In 2020, differences between HP seed treatments in seeding outcomes varied by site, species, and herbicide application for grasses and sagebrush, and the below results are given site-by-site for grasses first, then sagebrush below.

In ID, ELEL showed higher early seedling count of small HP pellet (4.0%) than both HP coatings, and higher seedling count for vortex (2.6%) than Kamterter coating (1.6%), with no differences between treatments for PSSP (F0.6 - 1.3%; SPEC*HPTRT interaction, F_3,90_ = 2.98, *P* = 0.0353). By later in the season, small HP pellet still had higher seedling count (2.2%) than both coatings (1.0-1.1%), which did not differ from one another (SPEC*HPTRT interaction; F_3,90_ = 3.12, *P* = 0.030). The effect of herbicide application on seedling count did not differ by seed treatment, with higher early seedling count in the presence of herbicide (1.7%) than the absence (1.2%), but lower later seedling count in the presence (0.3%) than the absence (1.2%; HERB main effects; F_3,90_ = 7.7 - 18.4, *P* < 0.007). For seedling size measurements, there was no difference between HP treatments for seedling height or leaf count for either species of grass.

In NV, there were no differences in seedling count among HP seed treatments for either species, early or late, and there was overall 34% lower late season seedling count in the presence than the absence of herbicide regardless of species or seed treatment (F_1,90_ = 11.1, *P* = 0.001). Additionally, there was no difference in height between HP coatings, and taller seedlings in the presence (45.2 mm) than the absence of herbicide (36.3 mm; HERB main effect; F_1,77_ = 9.54, *P* = 0.003), and number of leaves was higher for vortex coating (4.9) than Kamterter (3.2) or small HP pellet (3.4) in the presence of herbicide (with no such difference in the absence), and leaf number was generally higher in the presence (3.4 – 4.9) than the absence (2.3 – 2.9) of herbicide for all but Kamterter coatings (HERB*HPTRT interaction, F_3,77_ = 3.21, P = 0.0277).

In OR, small HP pellet had higher early season seedling count (5%) than coatings (3-3.5%) regardless of species or herbicide application (HPTRT main effect; F_3,90_ = 4.7, *P* = 0.004), but there were no differences among seed treatments by late season, which showed lower overall seedling count (1.3%) in the presence than the absence (2.7%) of herbicide (HERB main effect; F_1,90_ = 18.8, *P* < 0.001). Additionally, small HP pellet produced taller seedlings (43.2 mm) than either coating (33-34 mm; HPTRT main effect; F_3,72_ = 3.03, *P* = 0.0346), with no difference in leaf number among treatments, and ELEL seedlings were taller in the presence (43.8 mm) than the absence of herbicide (32.4 mm), with no other effects of herbicide on seedling size; SPEC*HERB interaction; F_1,72_ = 6.83, *P* = 0.0109).

In UT, small HP pellet had higher early seedling count (1.5%) than vortex coating (0.9%), with Kamterter coating intermediate (HPTRT main effect; F_3,90_ = 3.4, *P* = 0.021), but no differences remained among HP seed treatments by late season, and there was no effect of herbicide application either early or late. There were no differences between HP treatments in height or leaf number, and seedlings were in general taller (37.2 mm) and more leafy (3.4 per plant) in the presence than the absence (25.4, 3.03) of herbicide (HERB main effects; F_3,56_ = 4.3 - 19, *P* < 0.0418).

In WY, there were no differences in seedling count or seedling height or leaf count between HP treatments either early or late, and herbicide application had only early season effects, with lower seedling count in the presence (0.2%) than the absence (0.4%) of herbicide (HERB main effect; F_1,90_ = 4.21, *P* = 0.046).

For sagebrush, there were no differences in seedling count, early or late, between HP seed treatments at the only two sites with enough seedlings to analyze (OR, ID), regardless of other factors. Additionally, there were too few seedlings of sagebrush to evaluate any effects on seedling size. However, HP slurry was the only treatment to produce any seedlings at all in both OR and ID in both the presence and absence of herbicide, whereas HP pellet only produced seedlings at the ID site in the absence of herbicide.

**Performance of herbicide protection treatments compared to bare seed with and without herbicide – full results**

In the 2018 seeding, differences in seedling count between herbicide protection treatments and bare seed varied by grass species, herbicide application, and site in the early season. For POSE at the OR site, small and large HP pellets had 2.3 and 2.7-fold higher early seedling counts than bare seed, regardless of herbicide application (Fig 4; SPEC*HPTRT interaction; F_2,96_ = 11.7, *P* < 0.001). For POSE at both sites, these differences in seedling count were no longer present in late season data. There were no differences in seedling count between HP pellets and bare seed for ELEL at either site, early or late, regardless of herbicide application. For ARTRWY, both HP pellet sizes produced 48-68% lower early seedling count and 63-78% lower late seedling count than bare seed, regardless of whether herbicide was applied (SPEC*HPTRT main effects; F_2,96_ = 7.7-12.1, *P* < 0.001). The only difference in seedling height between bare seed and any size of HP pellet occurred in OR, with 1.6-fold taller plants for large pellets than bare seed, regardless of herbicide application (F_2,73_ = 5.7, *P* = 0.005). Differences in seeded species leaf counts between bare seed and HP pellets were seen only in OR, and only in the presence of herbicide, where they were higher for bare seed (6 per plant) than small HP pellets (3.4 per plant), regardless of species, with large pellets (4.7) intermediate (HERB*HPTRT interaction; F_2,73_ = 3.54, *P* = 0.034).

In the 2019 seeding, in the presence of herbicide, both pellet sizes produced 2.7-3-fold higher late season grass seedling count than bare seed, regardless of site or species, whereas neither pellet size differed from bare seed seedling count in the absence of herbicide (Fig 4; HPTRT*HERB interaction; F_2,240_ = 4.22, *P* = 0.0159). In OR, there was no difference in ARTR seedling count between either HP seed treatment and bare seed. The only differences in seedling size between HP treatments and bare seed were observed for ELEL and ARTR height in OR. For ELEL, both large and small pellets produced 1.9-fold taller seedlings than bare seed, in the presence, but not in the absence, of herbicide (HPTRT*HERB interaction, F_2,16_ = 5.49, *P* = 0.0153). For ARTR, carbon strip produced shorter seedlings (12.2 mm) than bare seed (5.8 mm) and small HPP (27.5 mm) in the presence of herbicide, with no such difference in the absence (HPTRT*HERB interaction, F_2,12_ = 3.91, *P* = 0.0493).

In the 2020 seeding, in ID, all three HP treatments produced higher early and late seedling count (1.0 – 4.0%) than bare seed (0.3 – 0.9%) for ELEL, and only small HP pellet produced higher seedling count (1.3%) than bare seed for PSSP (0.2%) in the early season (with no difference by late season), regardless of other factors (Fig 4; SPEC*HPTRT interactions,; F_3,90_ = 3.0 – 3.1 , *P* < 0.0353). In NV, the small HP pellet produced higher early ELEL seedling count (3.8%) than bare seed (2.1%) in the presence – but not absence - of herbicide (SPEC*HPTRT interaction; F_3,90_ = 2.74, *P* = 0.049), though no differences between bare seed and HP treatments remained in the late season. In OR, small HP pellet produced higher seedling count (5.0%) than bare seed (3.0%) early in the season regardless of other factors, with HP coatings intermediate (3.2-3.5%; HPTRT main effect; F_3,90_ = 4.72, *P* = 0.0042). In the late season, no differences among treatments remained, but seedling count was higher in the absence (2.7%) than the presence (1.4%) of herbicide (HERB main effect; F_1,90_ = 18.8, *P* < 0.001). In UT, bare seed and small HP pellet produced higher early seedling count (1.5-1.6%) than HP vortex coating (0.9%) regardless of other factors (with Kamterter intermediate; 1.1%), and bare seed produced higher late season seedling count (1.0%) than both HP coatings (0.04 - 0.05%), with small HP pellet intermediate (0.07%; HPTRT main effects; F_3,90_ = 3.0 - 3.4, *P* < 0.0337). No differences between bare seed and HP treatments were observed in WY.

Early ARTRWY seedling count was higher for the HP slurry (0.8%) than bare seed (0.2%) in the presence of herbicide (with HP pellet intermediate; 0.6%), regardless of site, with no such differences in the absence of herbicide (Fig 4; 0.3-0.7%; HERB*HPTRT interaction; F_2,66_ = 4.25, *P* = 0.0182). Later in the season, there were no differences in seedling count among treatments in ID, and in OR, bare seed seedling count (0.4%) was higher than both HP treatments (< 0.1%) in the absence of herbicide (SITE*HERB*HPTRT interaction; F_2,66_ = 3.88, *P* = 0.026), though HP slurry was the only treatment to produce seedlings in the presence of herbicide at either site.

There was only one site at which bare seed differed from HP treatments with respect to seedling size for grasses or sagebrush. In UT, bare seed treatment produced taller seedlings (34.8 mm) than HP pellet (29.7 mm) or vortex coating (26.2 mm), with Kamterter coating intermediate, regardless of other factors (HPTRT main effect; F_3,56_ = 3.15, *P* = 0.0320).

**Exceptions to the overall trend that the furrow treatment produced higher seedling counts and larger seedling sizes and broadcast seeding in 2018**

In OR, both early and late seeded species seedling count was higher for furrow (10.7% early, 2.8% late) than broadcast seeding (4.1% early, 0.8% late) regardless of treatment, though there was no difference between delivery methods for late-season POSE seedling count in the absence of herbicide (Fig 5; early DELIV main effect; F_1,96_ = 67.3, *P* < 0.001; late DELIV*SPEC*HERB interaction; F_1,96_ = 6.67, *P* = 0.0113). In NV, early POSE seedling count was higher in furrow (5.5%) than broadcast (1.1%) only in the presence of herbicide, and higher for ELEL in furrow (2.4%) than broadcast (1.1%) only in the absence of herbicide (SPEC*DELIV*HERB interaction; F_1,96_ = 5.54, *P* = 0.0205). In WY, furrow seeding ARTR produced higher early seedling count (7.4%) than broadcast (2.3%), regardless of all other factors, and higher late seedling count (3.7%) than broadcast seeding (0.8%) regardless of herbicide treatment, though large HP pellets in the late season showed no difference (0.06 – 1.6%) among seed delivery method (early DELIV main effect; F_1,96_ = 76.5, *P* < 0.001; late HPTRT*DELIV interaction; F_2,96_ = 5.86, *P* = 0.0040). There were no significant effects of seed delivery method on seedling height or leaf count in the OR site or WY sites, but, in NV, furrow seeding produced taller seedlings (26.3 mm) than broadcast (17.6 mm) for both species regardless of other factors (DELIV main effect; F_1,56_ = 4.67, *P* = 0.0350), and leafier plants (7.1 per plant) than broadcast (3.6 per plant) for both species in the presence (but not the absence) of herbicide (DELIV*HERB interaction; F_1,56_ = 7.81, *P* = 0.007).

**Effect of litter reduction**

The litter reduction treatment reduced litter depth at all sites, despite a significant SITE*LTR interaction (S4 Table 2 and 3). Additionally, litter reduction had no affect on IAG density or cover across sites or herbicide treatments, and the only effect on density or cover of IAF occurred in UT and OR, with litter reduction associated with higher IAF density in UT, and lower IAF cover in reduced litter than intact litter subplots exposed to herbicide in OR (S4 Table 4 & 5).

**S4 Table 2. ANOVA results for litter reduction treatment on litter depth**

| **2019 litter depth** | **Nparm** | **DF** | **Sum of Squares** | **F Ratio** | **Prob > F** |
| --- | --- | --- | --- | --- | --- |
| Site | 4 | 4 | 4097.090 | 13.0002 | <.0001* |
| Litter | 1 | 1 | 11546.682 | 146.5515 | <.0001* |
| Herbicide | 1 | 1 | 759.186 | 9.6357 | 0.0026* |
| Site*Litter | 4 | 4 | 2223.547 | 7.0554 | <.0001* |
| Site*Herbicide | 4 | 4 | 575.176 | 1.8250 | 0.1323 |
| Litter*Herbicide | 1 | 1 | 159.927 | 2.0298 | 0.1582 |
| Site*Litter*Herbicide | 4 | 4 | 461.009 | 1.4628 | 0.2215 |

**S4 Table 3. Tukey HSD results for significant interaction involving litter reduction treatment on litter depth.**

| **2019 litter depth**  **Site*Litter interaction** |  |  |  |  |  | **Least Sq Mean** |
| --- | --- | --- | --- | --- | --- | --- |
| WY,Unraked | A |  |  |  |  | 45.10 |
| WY,Raked |  |  |  | D | E | 11.20 |
| UT,Unraked |  |  | C | D |  | 18.00 |
| UT,Raked |  |  |  |  | E | 6.50 |
| OR,Unraked | A |  |  |  |  | 43.42 |
| OR,Raked |  |  |  | D | E | 11.40 |
| NV,Unraked |  | B | C |  |  | 21.80 |
| NV,Raked |  |  |  |  | E | 8.80 |
| ID,Unraked |  | B |  |  |  | 26.70 |
| ID,Raked |  |  |  |  | E | 9.00 |

**S4 Table 4. ANOVA results for litter reduction treatment on IAG (top) and IAF (bottom) density (left) and cover (right).**

|  |  |  | **2019 IAG density** | | | **2019 IAG cover** | | |
| --- | --- | --- | --- | --- | --- | --- | --- | --- |
|  | **Nparm** | **DF** | **Sum of Squares** | **F Ratio** | **Prob > F** | **Sum of Squares** | **F Ratio** | **Prob > F** |
| Site | 4 | 4 | 863.4516 | 9.7585 | <.0001* | 181.03545 | 19.7901 | <.0001* |
| Litter | 1 | 1 | 4.5011 | 0.2035 | 0.6531 | 1.94733 | 0.8515 | 0.3589 |
| Herbicide | 1 | 1 | 2495.7257 | 112.8239 | <.0001* | 626.91799 | 274.1295 | <.0001* |
| Site*Lit | 4 | 4 | 123.6408 | 1.3974 | 0.2424 | 5.36029 | 0.5860 | 0.6737 |
| Site*Herb | 4 | 4 | 479.8364 | 5.4230 | 0.0006* | 33.05025 | 3.6129 | 0.0093* |
| Litter*Herb | 1 | 1 | 68.6744 | 3.1046 | 0.0819 | 9.00184 | 3.9362 | 0.0507 |
| Site*Lit*Herb | 4 | 4 | 131.2910 | 1.4838 | 0.2149 | 8.45927 | 0.9247 | 0.4539 |
|  |  |  | **2019 IAF density** | | | **2019 IAF cover** | | |
| Site | 4 | 4 | 123.44859 | 12.4635 | <.0001* | 45.602162 | 22.1732 | <.0001* |
| Litter | 1 | 1 | 3.52017 | 1.4216 | 0.2367 | 0.451148 | 0.8774 | 0.3517 |
| Herbicide | 1 | 1 | 20.57668 | 8.3098 | 0.0051* | 2.702632 | 5.2564 | 0.0245* |
| Site*Lit | 4 | 4 | 45.02808 | 4.5461 | 0.0023* | 5.368451 | 2.6103 | 0.0415* |
| Site*Herb | 4 | 4 | 106.44518 | 10.7469 | <.0001* | 30.236906 | 14.7021 | <.0001* |
| Litter*Herbi | 1 | 1 | 5.52407 | 2.2309 | 0.1392 | 0.674778 | 1.3124 | 0.2554 |
| Site*Lit*Herb | 4 | 4 | 22.34499 | 2.2560 | 0.0703 | 7.523847 | 3.6583 | 0.0087* |

**S4 Table 5. Tukey HSD results for significant interactions involving litter reduction treatment on IAF density (top) and cover (bottom).**

| **2019 IAF density**  **Site*Lit interaction** |  |  |  |  | **Least Sq Mean** |
| --- | --- | --- | --- | --- | --- |
| WY,Unraked |  |  |  | D | -4.441e-16 |
| WY,Raked |  |  |  | D | -8.882e-16 |
| UT,Unraked |  | B | C | D | 1.378504 |
| UT,Raked | A |  |  |  | 4.382494 |
| OR,Unraked |  |  | C | D | 1.280264 |
| OR,Raked |  |  | C | D | 1.169464 |
| NV,Unraked |  | B |  |  | 2.702984 |
| NV,Raked |  | B | C |  | 1.914946 |
| ID,Unraked |  |  |  | D | 0.428943 |
| ID,Raked |  |  |  | D | 0.200000 |
| **2019 IAF cover - Site*Lit*Herb interaction** | | | | | |
| WY,Unraked,N |  |  |  | D | 8.3267e-17 |
| WY,Unraked,H |  |  |  | D | -8.327e-17 |
| WY,Raked,N |  |  |  | D | -8.327e-17 |
| WY,Raked,H |  |  |  | D | 8.3267e-17 |
| UT,Unraked,N |  |  | C | D | 0.848528 |
| UT,Unraked,H |  |  | C | D | 0.882843 |
| UT,Raked,N |  |  | C |  | 1.372741 |
| UT,Raked,H |  | B |  |  | 2.429537 |
| OR,Unraked,N |  |  |  | D | 0.289443 |
| OR,Unraked,H |  |  | C |  | 1.315298 |
| OR,Raked,N |  |  | C | D | 0.680574 |
| OR,Raked,H |  |  |  | D | 0.334164 |
| NV,Unraked,N | A |  |  |  | 3.436145 |
| NV,Unraked,H |  |  |  | D | 1.1102e-16 |
| NV,Raked,N |  | B |  |  | 2.415748 |
| NV,Raked,H |  |  | C | D | 0.882843 |

**Germination**

Cumulative germination, as a percent of viable bare seed sown, varied among species and between sites within species, but no statistical comparisons are available (S5 Fig). Comparisons across years are not suitable, due to different seed lots each year (S1 Appendix). In 2018, neither ARTR nor ELEL showed cumulative germination of viable seed over 50% at any site, whereas this mark was surpassed by all species in at least two sites in 2019 and 2020. Overall, ARTR showed less pre-winter germination than ELEL and POSE (this was unmeasured for PSSP). Across species and years, emergence of sown, viable seed was between 0 to 5% of the total germination rate, with the exception of sagebrush in WY in 2018, which was 25%.

**S5 Fig. Germination timing of bare seed.** Germination of bare seed (placed in shallow seed bags) as a percent of estimated viable seed sown, by species (across top) and planting year (top to bottom). The height of each bar represents the mean cumulative germination of viable seed sown for each site, year, and species, with the portion of this total that occurred within each harvest period indicated by different colors. Asterisks note instances of cumulative germination that exceed 100% of estimated viable seed sown, which suggests field conditions encouraged higher germination rates than petri dish tests used to develop the estimates of viable seed sown. A pre-winter harvest was not made in seeding year 2020, so the pre-spring harvest contains all pre-winter and winter germination for that year.
