# Supplementary figures and images for: Assessment of multiple herbicide protection seed treatments for seed-based restoration of native perennial bunchgrasses and sagebrush across multiple sites and years
Source: PLoS One. 2023 Mar 30;18(3):e0283678. doi: 10.1371/journal.pone.0283678 (PMC10062626; doi:10.1371/journal.pone.0283678)

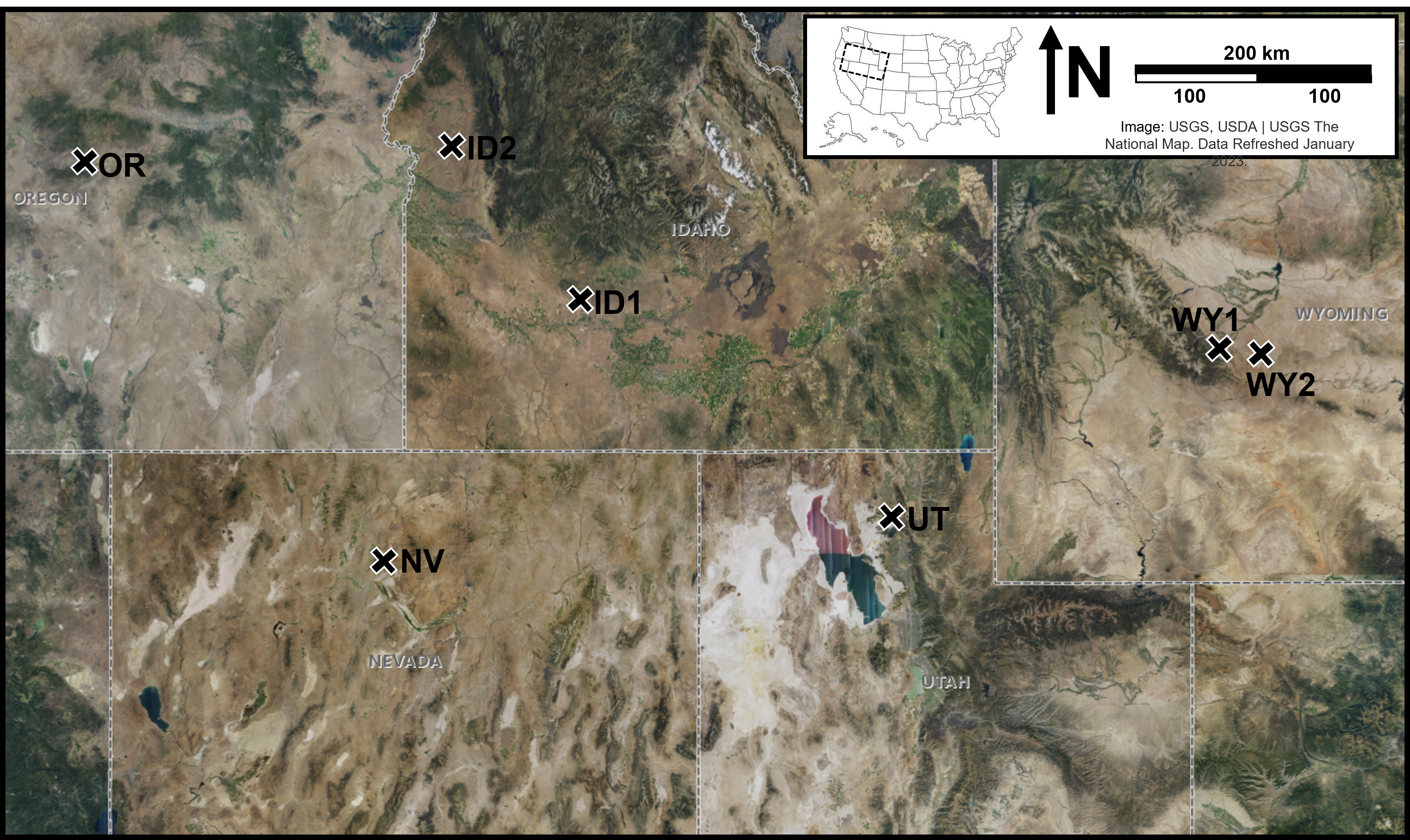

Supplement: S1 Fig — Site names correspond to tables. In Idaho and Wyoming, original sites (1) were relocated to improved sites (2), and trials were never carried out in both sites in the same year within a state. (TIF) [file pone.0283678.s003.tif]

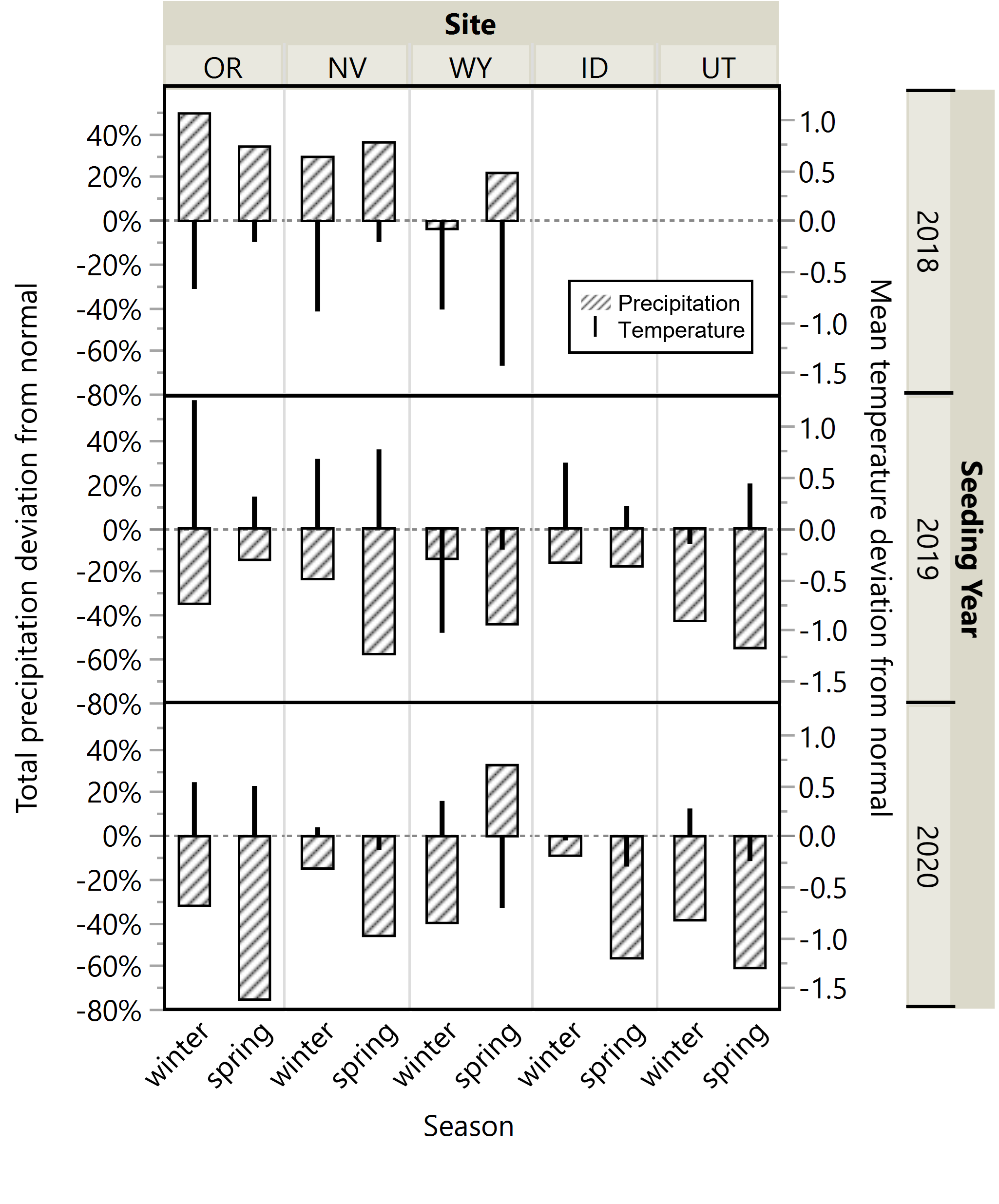

Supplement: S2 Fig — Mean temperature anomaly (black needles) and precipitation anomaly (hashed bars) for all tested sites (across top) and planting years (2018, 2019, 2020; top to bottom), for winter (Dec–Feb) and spring (Mar–May) seasons, calculated against 1991–2020 climate normals. Values are percent deviation from the normal precipitation (left axis) and degrees Celsius (right axis). Data are from PRISM Climate Group. (TIF) [file pone.0283678.s004.tif]

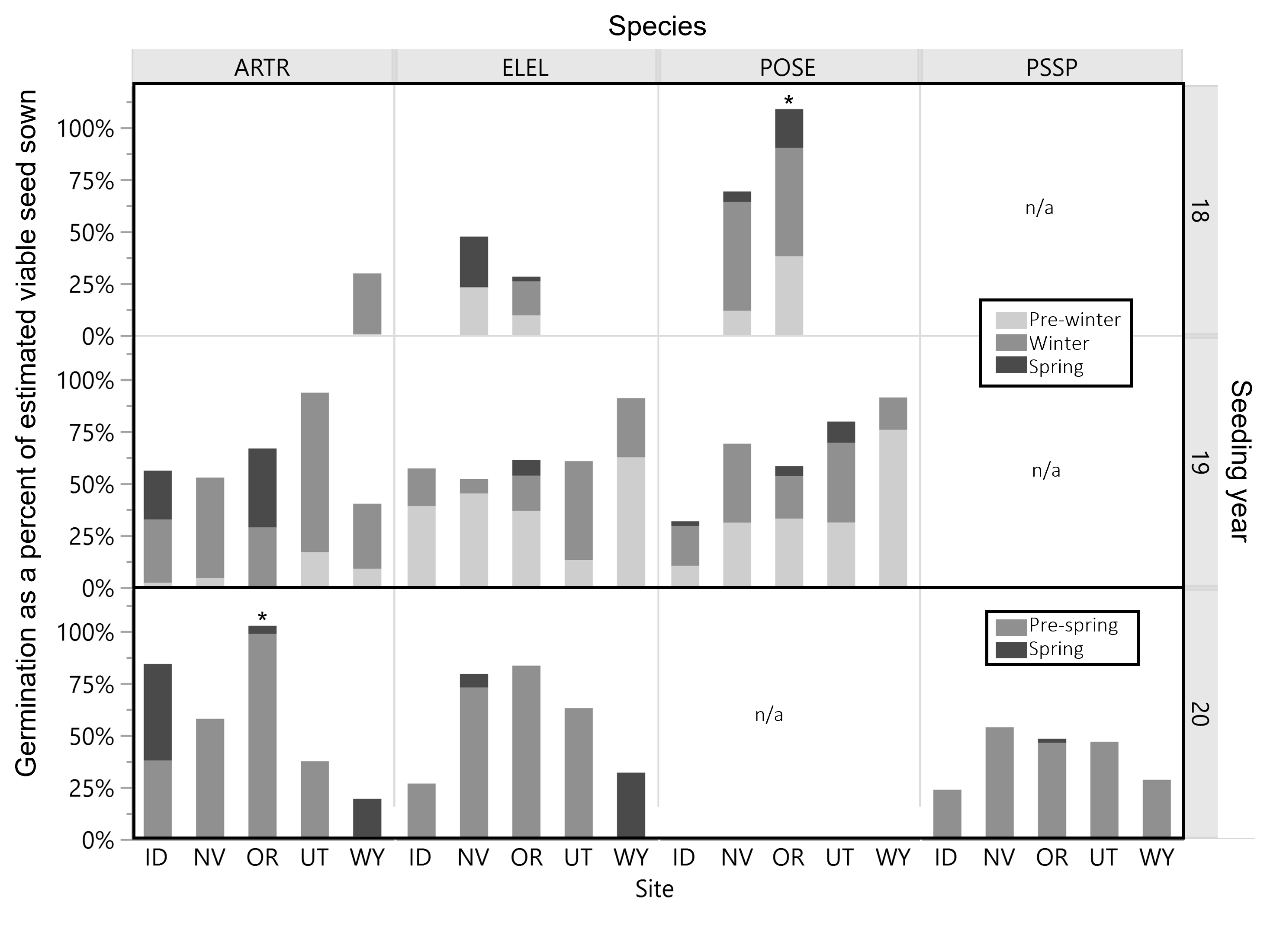

Supplement: S3 Fig — Germination of bare seed (placed in shallow seed bags) as a percent of estimated viable seed sown, by species (across top) and planting year (top to bottom). The height of each bar represents the mean cumulative germination of viable seed sown for each site, year, and species, with the portion of this total that occurred within each harvest period indicated by different colors. Asterisks note instances of cumulative germination that exceed 100% of estimated viable seed sown, which suggests field conditions encouraged higher germination rates than petri dish tests used to develop the estimates of viable seed sown. A pre-winter harvest was not made in seeding year 2020, so the pre-spring harvest contains all pre-winter and winter germination for that year. (TIF) [file pone.0283678.s005.tif]

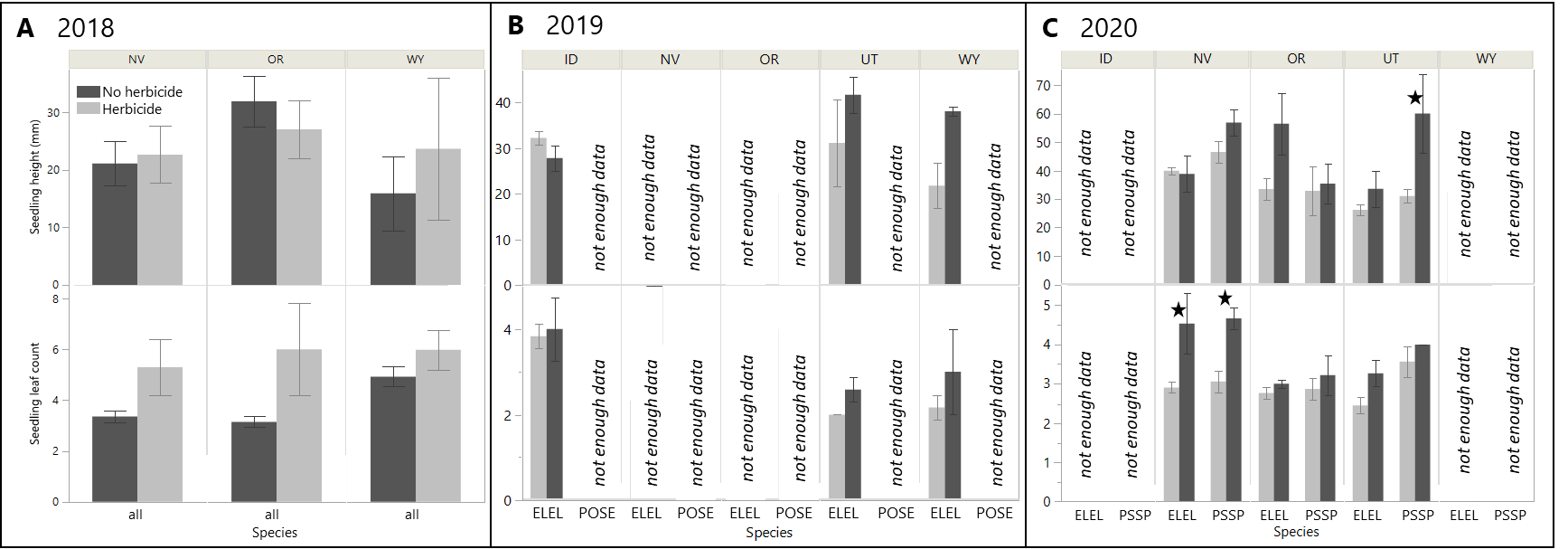

Supplement: S4 Fig — Effect of herbicide on mean seedling height (mm) and mean seedling leaf count of seedlings derived from the bare seed (unprotected) seed treatment for all three years. In 2018 (A), all species and both delivery methods (broadcast, furrow) are pooled. In 2019 and 2020 (B), only furrow delivery data are included. Black stars indicate significant effect (P < 0.05), and all other comparisons are not significant. Error bars are standard errs. Too few seedlings for some species in some years resulted in not enough data to make statistical comparisons. (TIF) [file pone.0283678.s006.tif]

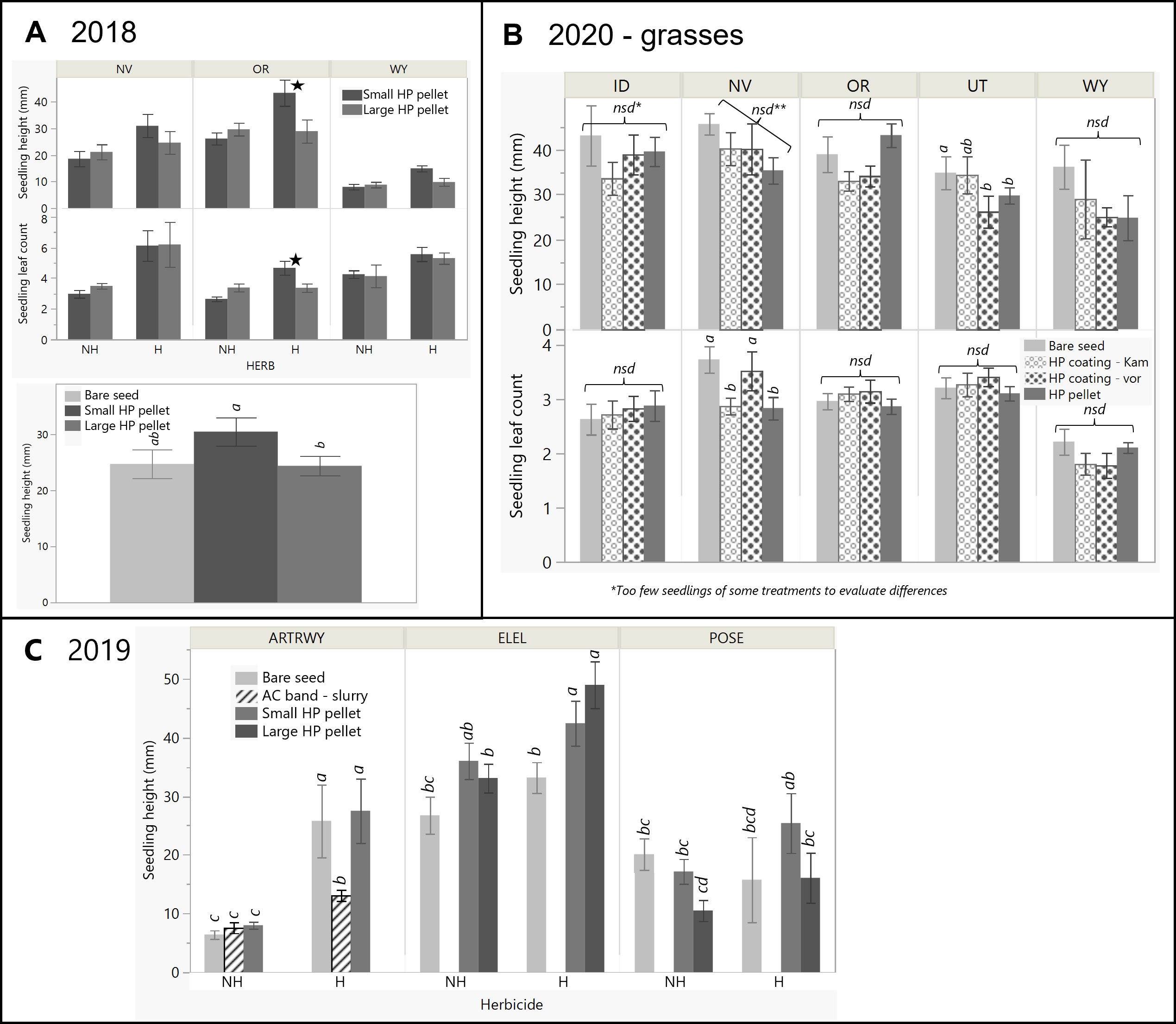

Supplement: S5 Fig — Differences in seedling size (mean height and leaf count) among seed treatments. In 2018 (A), differences among carbon seed treatments were dependent upon exposure to herbicide (top), but notable differences between carbon treatments and bare seed were not (bottom). Black stars indicate significant difference in ANOVA model (P < 0.05). Bars sharing the same letters within each site for 2020 (B) and for each species in 2019 (C) are not significantly different according to post-hoc Tukey HSD tests (P < 0.05). (TIF) [file pone.0283678.s007.tif]
