# Supplementary material for: Assessment of multiple herbicide protection seed treatments for seed-based restoration of native perennial bunchgrasses and sagebrush across multiple sites and years
Source: PLoS One. 2023 Mar 30;18(3):e0283678. doi: 10.1371/journal.pone.0283678 (PMC10062626; doi:10.1371/journal.pone.0283678)
Supplement: Attachment — Submitted filename: Response to Reviewers.docx [file pone.0283678.s009.docx]

*Hello,*

*Thank you for the very timely review and the provisional acceptance of our work, dependent upon revision. We appreciate opportunity to make the below changes (our responses in italic and intended) to our manuscript. We believe these changes satisfy all requested changes but will gratefully address additional requests if needed.*

*Sincerely,*

*Owen Baughman (on behalf of all authors)*

Changes to the manuscript in response to in-line edits by the reviewer:

*All instances of AC (activated carbon) have been replaced with HP (herbicide protection) to be consistent, because it is clear that our HP treatments rely entirely on AC as the active ingredient.*

*Misspelling in Table 2 (surface) corrected (surface).*

*The typo “vibility” in table S1 was corrected, thank you.*

*Instances of AC reference in Table 3 and footnotes were changed to HP to maintain consistency with text.*

*Abbreviation of “Kam” in Table 3 changed to “Kamterter” to be consistent with text.*

*Typo of “acre” in Table 3 footnotes corrected to hectare (“ha”)*

*Typo of species (ARTRWY) corrected in Table 3 footnotes (ARTR).*

*Space added to “seed lot” in Table 3 footnote.*

*Instance of repeated phrase at line 500 was reworded.*

*Space added at line 637 to correct typo*

Reviewer comment at line 501: “You say “improvement in seeding success” above…”

*We will keep the current wording (“reduction in seeding success”) because if the seed technologies perform more poorly than bare seed, that is not an ‘improvement’ in success of bare seed over the seed technologies, because the bare seed is the baseline for comparison. Therefore, referring to it as a reduction in success for the seed technologies is an accurate way to refer to this difference. We are happy to reconsider if we have misinterpreted your comment.*

Editor comments:

1.  Please ensure that your manuscript meets PLOS ONE's style requirements, including those for file naming.

*We reviewed the Style Guide and corrected the names of supporting information files and corrected a few cels in table to remove “returns” used for spacing. We believe the rest of our submission adheres to the style guide but will be happy to make additional corrections if we have missed something.*

2. In your Methods section, please provide additional information regarding the permits you obtained for the work. Please ensure you have included the full name of the authority that approved the field site access and, if no permits were required, a brief statement explaining why.

*Thank you for pointing out this omission. We have added wording (line 129-130) explaining the permits obtained for the three sites that needed it, and explaining that the remainder of the sites did not require formal permitting.*

3. We note that S2 Fig in your submission contain [map/satellite] images which may be copyrighted.

*Thank you for spotting this, our apologies for this oversight. We have reworked S2 fig to contain public domain imagery from the USGS link provided.*

4. Please review your reference list to ensure that it is complete and correct. If you have cited papers that have been retracted, please include the rationale for doing so in the manuscript text, or remove these references and replace them with relevant current references.

*We have double checked our references and compared them against Retraction Watch’s list of retractions. We found several systemic format errors that we corrected (space after “doi:”, “from” after “Available”), corrected several typos, and found no instances of references to retracted works. We found no other issues with the references and in-text citations, but will be happy to address any that we have missed.*
